# Spatial Coherence Adaptive Clutter Filtering in Color Flow Imaging—Part I: Simulation Studies

**DOI:** 10.1109/ojuffc.2022.3184914

**Published:** 2022-06-21

**Authors:** WILL LONG, DAVID BRADWAY, RIFAT AHMED, JAMES LONG, GREGG E. TRAHEY

**Affiliations:** 1 Philips, Cambridge, MA 02141 USA; 2 Department of Biomedical Engineering, Duke University, Durham, NC 27708 USA; 3 Department of Radiology, Duke University Medical Center, Durham, NC 27710 USA

**Keywords:** Acoustic clutter, adaptive clutter filtering, color flow imaging, image quality, spatial coherence, ultrasound

## Abstract

The appropriate selection of a clutter filter is critical for ensuring the accuracy of velocity estimates in ultrasound color flow imaging. Given the complex spatio-temporal dynamics of flow signal and clutter, however, the manual selection of filters can be a significant challenge, increasing the risk for bias and variance introduced by the removal of flow signal and/or poor clutter suppression. We propose a novel framework to adaptively select clutter filter settings based on color flow image quality feedback derived from the spatial coherence of ultrasonic backscatter. This framework seeks to relax assumptions of clutter magnitude and velocity that are traditionally required in existing adaptive filtering methods to generalize clutter filtering to a wider range of clinically-relevant color flow imaging conditions. In this study, the relationship between color flow velocity estimation error and the spatial coherence of clutter filtered channel signals was investigated in Field II simulations for a wide range of flow and clutter conditions. This relationship was leveraged in a basic implementation of coherence-adaptive clutter filtering (CACF) designed to dynamically adapt clutter filters at each imaging pixel and frame based on local measurements of spatial coherence. In simulation studies with known scatterer and clutter motion, CACF was demonstrated to reduce velocity estimation bias while maintaining variance on par with conventional filtering.

## I. INTRODUCTION

Color flow imaging is a widely used technique to characterize blood flow and tissue motion in ultrasound. Its applications span from the measurement of hemodynamics for the assessment of peripheral artery and cardiovascular disease [1], [2] to the characterization of ventricular wall [3] and valve [4] function in echocardiography. Despite its widespread use, color flow imaging is known to suffer from artifacts generated by clutter. In the Doppler literature, clutter refers to unwanted echoes from slow-moving vessel walls and tissue that superimpose onto low amplitude blood signal and interfere with blood velocity estimation. These unwanted echoes can include acoustic noise generated by multi-path reflections and off-axis scattering. Clinically, clutter leads to velocity estimation bias that obscures the visualization of clinically-relevant flow patterns and decreases the reliability of measurements used in the assessment of disease [5]–[7].

In conventional color flow imaging, this bias is reduced by means of a high-pass filter, commonly referred to as a clutter or wall filter, which functions to attenuate stationary or slowly moving signals and preserve higher velocity blood and/or tissue. These filters, which are typically finite or infinite impulse response (FIR or IIR) in design, have fixed frequency responses, limiting their ability to generalize to different imaging conditions. Given the complex spatio-temporal dynamics of tissue, blood, and clutter, the optimal filter and parameters which best isolate flow signal can vary significantly between different imaging windows, vessels, and even pixels. Consequently, the quality of conventional color flow imaging is associated with a high degree of operator-dependence, relying heavily on the appropriate selection of clutter filters by sonographers and the careful design of filter presets by manufacturers [5], [8].

To reduce the operator dependence of color flow imaging, several techniques have been proposed to adapt clutter filtering based on imaging feedback. These methods generally fall into two categories: 1) adaptive methods measuring tissue motion and 2) eigen-based filtering. Methods for adaptive down-mixing [9]–[11] and filter selection [12]–[14] adapt to tissue motion by either down-mixing measured slow-time signals (samples across emissions sampled at the pulse repetition frequency) or by selecting filters based on measurements of mean tissue frequency. In this way, slow-time signals or clutter filtering can be dynamically modulated to optimally suppress moving tissue and any clutter coupled with it. In eigen-based filtering, rather than relying on direct measurements from tissue, slow-time signals are decomposed through methods such as eigenvalue decomposition (EVD) [10], [15], singular value decomposition (SVD) [16]–[18], principal component analysis (PCA) and independent component analysis (ICA) [19] to adaptively identify and remove clutter based on the magnitude and frequency of individual slow-time components.

Although existing methods demonstrate promise to adapt clutter filtering to a broader range of flow conditions, they rely on a number of fundamental assumptions limiting their ability to enable fully adaptive, user-independent color flow imaging. Methods for adaptive down-mixing and filter selection assume that clutter motion is well characterized by measurements of tissue motion, which is not always the case. For example, reverberation clutter motion is dictated by the source of multi-path scattering which is often spatially distinct and moving at different velocities relative to tissue within the imaging field-of-view [20], [21]. A similar phenomenon is expected for off-axis clutter arising from distant, but highly echogenic structures. Both scenarios represent failure modes in the existing methods for adaptive down-mixing and filter selection, where local estimates of tissue motion poorly capture clutter motion, resulting in decreased clutter suppression.

Eigen-based filtering methods are similarly limited by the assumptions required to identify the eigen-components corresponding to clutter. Such techniques generally employ magnitude and frequency thresholds, under the assumption that blood signal is lower magnitude and higher velocity than tissue or clutter. These thresholds restrict the range of measurable signals, imposing limits on the minimum detectable velocity and the ability to simultaneously measure velocities from both high and low magnitude signals. The ability to separate eigen-components in magnitude and frequency space is furthermore highly dependent on ensemble length and degrades with the short ensembles used in conventional color flow imaging (typically 8–16 samples) [10], [15], [22].

We can see from the existing methods in adaptive clutter filtering that magnitude and frequency information is often insufficient to fully separate flow from clutter in many of the complex imaging conditions encountered *in vivo*. In an attempt to mitigate this deficiency, this study explores the application of backscatter spatial coherence as a feedback metric to replace or augment the magnitude and frequency information already leveraged by existing adaptive clutter filtering techniques. Herein, we use spatial coherence to refer to the aperture domain coherence, i.e. the similarity of backscattered echoes received across individual elements of an ultrasound array. Studies have shown a direct relationship between measurements of spatial coherence and local clutter level with behavior that is well-predicted by theory [23]–[25]. This relationship has been successfully leveraged in numerous applications including the adaptive selection of acoustic output in harmonic imaging [25], aberration correction [26], [27], coherence beamforming [28]–[30], coherent flow power Doppler (CFPD) imaging [31], among many others. In the context of adaptive clutter filtering, the spatial coherence of post-clutter filtered signals can be used to iteratively adjust clutter filter settings to attenuate clutter from off-axis interference and reverberation, which decrease coherence, and pass through on-axis signals from blood and tissue, which increase coherence. Such a mechanism shows promise as a means to better adapt clutter filtering under conditions where methods relying on the magnitude and frequency of signal and clutter break down.

To evaluate the application of spatial coherence image quality feedback in adaptive clutter filtering, Section II-A outlines the methods used to simulate color flow channel data and moving clutter. These simulations are applied to examine the spatial coherence of clutter filtered channel data in Section II-B and the relationship between spatial coherence and color flow image quality in Section II-C. In Section II-D, we describe an implementation of coherence-adaptive clutter filtering or CACF designed to maximize the spatial coherence of clutter filtered slow-time signals at each imaging location and in each frame of color flow data. This method is evaluated in a number of simulation studies with regards to its dependence on coherence imaging resolution and texture in Section III-B and its performance relative to conventional clutter filters in Section III-C. This is followed by a discussion of the limitations and future directions in Section III-E with conclusions in Section IV.

## II. METHODS

### A. SIMULATION OF COLOR FLOW CHANNEL DATA

Channel data were simulated in Field II [32], [33] for a series of 1-cm diameter vessels imaged using a 128-element C5–2v curvilinear array transmitting and receiving 4-cycle pulses at 3.5 MHz with a 6 cm focus and F/2 focal geometry. Simulations were performed for multiple transmit-receive cycles to generate color flow ensemble data consisting of 14 slow-time samples captured at a 3 kHz pulse repetition frequency (PRF), corresponding to a 33 cm/s Nyquist velocity.

#### 1) FLOW SIMULATION

**FIGURE 1.**
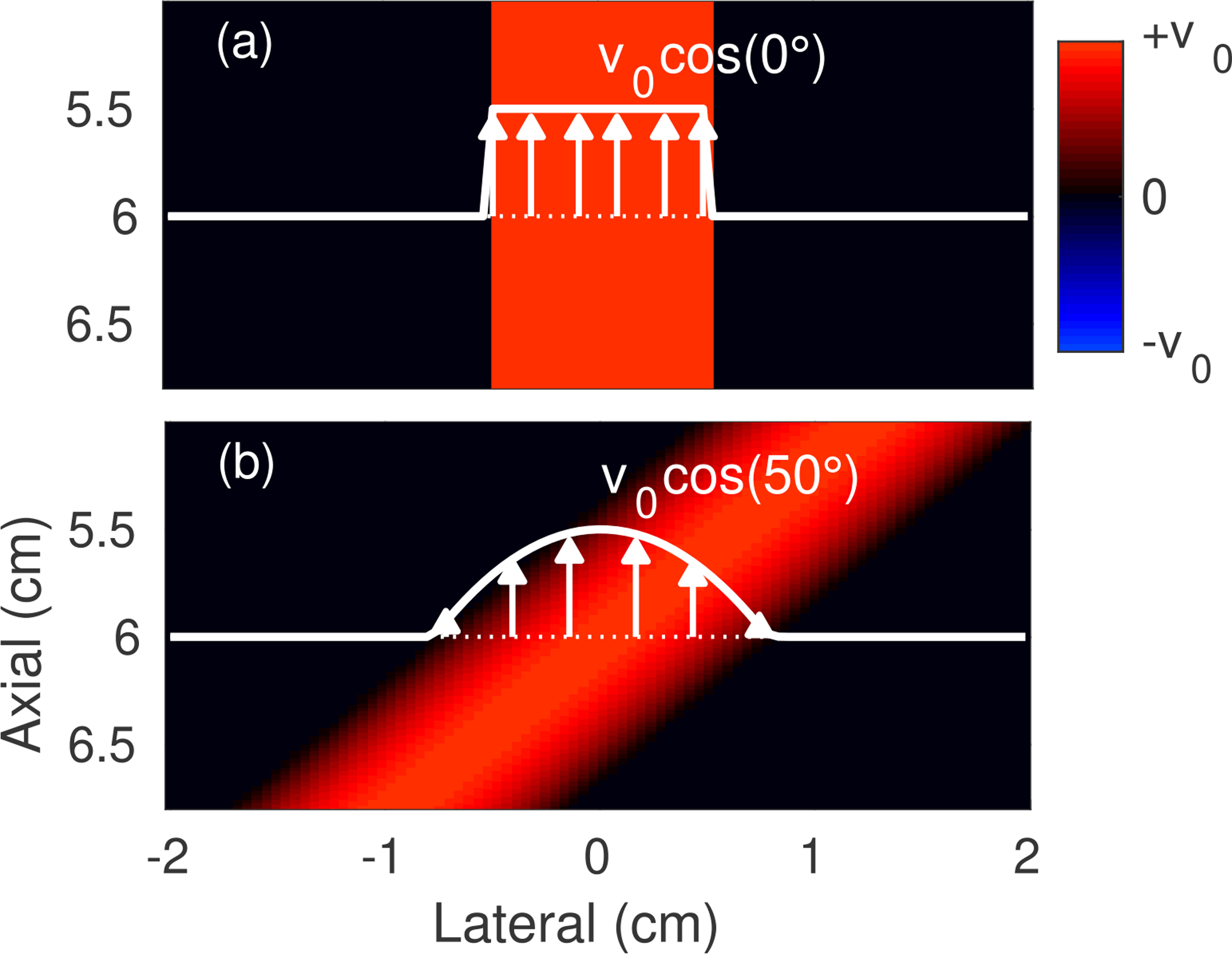
Simulated velocities for (a) 0° plug and (b) 50° parabolic flow. Profiles of the axial velocity as a function of lateral location at the 6 cm focal depth are shown in white.

Simulations of different blood vessels were performed using a 40 × 40 × 1 mm block populated with 15 scatterers per resolution cell. The backscatter amplitude of blood inside each vessel was set to −40 dB relative to surrounding background tissue scatterers. To simulate flow, blood scatterers within each 1-cm diameter vessel were displaced between transmit-receive cycles with a prescribed motion based on their spatial location. Velocity fields (Fig. 1) were simulated for both 0° plug and 50° parabolic flow with peak axial velocities *v*_0_ ranging from 3 to 18 cm/s.

#### 2) THERMAL NOISE AND ACOUSTIC CLUTTER MODELS

To model thermal noise, Gaussian white noise with varying signal-to-noise ratio was added to individual channel signals. This channel signal-to-noise ratio is termed SNR_*c,t*_ below, where *c* and *t* denote channel and thermal noise, respectively, and this ratio is calculated relative to the channel signal power of blood. Additionally, simulations were performed to study acoustic clutter from both spatially incoherent noise and off-axis scattering from surrounding tissue.

- **Spatially Incoherent Clutter:** Sequential transmissions at the same location, with propagation through the same realization of tissue wall, will result in the same echo contributions from multi-path reverberation. In the absence of tissue motion, clutter arising from these reverberations or rapidly spatially varying aberrations can be described as being uncorrelated between channel signals (i.e. spatially incoherent), but temporally stable and coherent across slow-time [21], [34]. Such characteristics are fundamentally distinct from thermal noise, which is both uncorrelated and random along both dimensions of time and space. Spatially incoherent clutter is modeled in this study using additive Gaussian white noise that is filtered by the transducer bandwidth and uncorrelated between channels, but constant across the slow-time samples in the absence of motion. In this way, the correlation of noise is *ρ*_*c*_ = 0 between channels and *ρ*_*t*_ = 1 between slow-time samples collected at the same channel. In this study, spatially incoherent and temporally coherent channel noise (N_*c,s*_ where *c* and *s* denote channel and spatial noise, respectively) was added from −10 to 10 dB power relative to the channel signal power of blood echoes, i.e. SNR_*c,s*_ = −10 to 10 dB. To simulate moving clutter that is common under *in vivo* conditions with transducer and physiological motion, this static channel noise was displaced at constant velocities ranging from −3 to 3 cm/s and subsequently added to the channel data generated by Field II simulations of each blood vessel.
- **Off-Axis Clutter:** In addition to spatially incoherent clutter, bulk motion can also result in the leakage of partially coherent clutter from off-axis scattering into the filter passband. To simulate bulk motion and the effects of off-axis clutter introduced by bulk motion, the velocity fields described in Section II-A1 were first applied to simulate blood flow followed by a bulk displacement of the entire 4×4 cm block containing both blood and tissue scatterers. Simulations were performed for a range of bulk velocities from 0 to 3 cm/s along the axial direction in the direction of blood flow.

### B. SPATIAL COHERENCE OF COLOR FLOW CHANNEL SIGNALS

**FIGURE 2.**
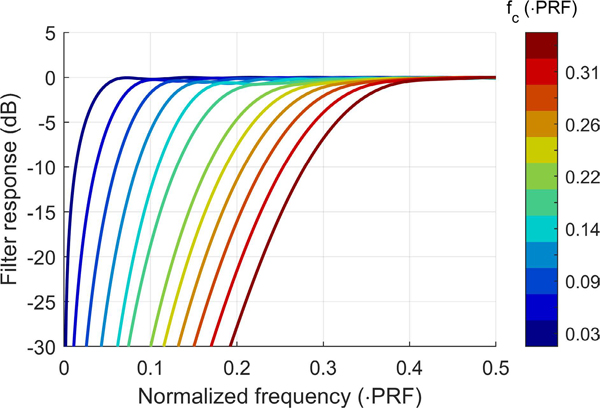
Frequency responses of each projection-initialized IIR clutter filter used in this study plotted with increasing cutoff frequency from blue to red.

To examine the spatial coherence of clutter filtered backscatter, simulated channel data were passed through 12 unique projection-initialized infinite impulse response (PI-IIR) clutter filters implemented based on methods presented in [22]. Filters were designed with a range of −3 dB cutoff frequencies (*f*_*c*_) from 0.03 to 0.34 · PRF (90 Hz to 1.02 kHz) corresponding to velocity cutoffs (*v*_*c*_) from 2.0 to 22.4 cm/s. The frequency responses of all 12 PI-IIR filters are shown in Fig. 2.

Spatial coherence functions at the output of each filter were calculated as the normalized cross-correlation of clutter filtered channel signals averaged as a function of element spacing, or spatial lag *m*, and across the slow-time ensemble:

(1) $$\hat{R}[m;\;\vec{r}]=\frac{1}{K(M\;-\;m)}\sum_{k=1}^{K}\sum_{i=1}^{M-m}\frac{u_{i}[\vec{r},\;k]u_{i+m}^{*}[\vec{r},\;k]}{\left|u_{i}[\vec{r},\;k]\right|\left|u_{i+m}[\vec{r},\;k]\right|}$$

where *M* is the total number of receive elements, *u*_*i*_ and *u*_*i*+*m*_ are time-delayed complex in-phase and quadrature (IQ) channel signals received at elements *i* and *i*+*m*. This expression is similar to those used for computing spatial coherence in conventional imaging [26], [35], but adds an averaging operation across the ensemble of coherence values obtained from each slow-time sample *k* to collapse the output at each imaging location $\vec{r}$ into a 1-D coherence function $\hat{R}[m]$.

### C. SPATIAL COHERENCE AND COLOR FLOW IMAGE QUALITY

As in conventional B-mode imaging, measurements of the spatial coherence can be similarly related to image quality in color flow imaging. To examine this relationship, color flow velocity estimates were derived via 2D autocorrelation [36] (2) and (3), as shown at the bottom of the page, which calculates the average axial velocity between in-phase (I) and quadrature (Q) beamsum signals *k* and *k* + 1 across an ensemble of *K* slow-time samples for a kernel $\Delta \vec{r}$ centered about imaging location $\vec{r}$ given speed of sound *c*, pulse repetition frequency PRF, and center frequency *f*_0_.

Color flow image quality was quantified via percent bias and standard deviation of velocity estimates, as defined in [37]:

(2) $$\hat{\phi }[\vec{r}]=\tan^{-1}\left(\frac{\sum_{k=1}^{K-1}\sum_{r\in \Delta \vec{r}}Q[r,\;k\;+\;1]I[r,\;k]\;-\;Q[r,\;k]I[r,\;k\;+\;1]}{\sum_{k=1}^{K-1}\sum_{r\in \Delta \vec{r}}I[r,\;k\;+\;1]I[r,\;k]\;+\;Q[r,\;k\;+\;1]Q[r,\;k]}\right)$$

(3) $$\hat{v}[\vec{r}]=-\frac{c\text{PRF}}{4\pi f_{0}}\hat{\phi }[\vec{r}],$$

(4) $$Bias=\frac{1}{\max\{|v|\}}\frac{1}{N_{p}N_{r}}\sum_{i=1}^{N_{p}}\sum_{j=1}^{N_{r}}\left|\hat{v}\left[{\vec{r}}_{j},\;i\right]-v\left[{\vec{r}}_{j}\right]\right|,$$

(5) $$S.D.=\frac{1}{\max\{|v|\}}\sqrt{\frac{1}{N_{p}N_{r}}\sum_{i=1}^{N_{p}}\sum_{j=1}^{N_{r}}{\left(\hat{v}\left[{\vec{r}}_{j},\;i\right]-\bar{v}\left[{\vec{r}}_{j}\right]\right)}^{2}},$$

where

(6) $$\bar{v}\left[{\vec{r}}_{j}\right]=\frac{1}{N_{p}}\sum_{i=1}^{N_{p}}\hat{v}\left[{\vec{r}}_{j},i\right].$$

*N*_*p*_ is the number of lateral velocity profiles, *N*_*r*_ is the number of velocity estimates in each profile, ${\vec{r}}_{j}$ is the position of each sample *j* in the profile, $v\left[{\vec{r}}_{j}\right]$ is the ground truth axial velocity, $\hat{v}\left[{\vec{r}}_{j}\right]$ is the measured axial velocity, and $\bar{v}\left[{\vec{r}}_{j}\right]$ is the average across all profiles *i*. These metrics were summarized using a more general measure of velocity estimation error:

(7) Error=max{Bias,S.D.},

to characterize the maximum percent error due to either bias or standard deviation.

These metrics were then compared to corresponding measurements of the short-lag spatial coherence (SLSC):

(8) $$\mathrm{SLSC}[\vec{r}]=\sum_{m=1}^{Q}\hat{R}[m;\;\vec{r}],$$

which captures the integrated coherence function up to a maximum short-lag value *Q* [28].

### D. COHERENCE-ADAPTIVE CLUTTER FILTERING (CACF)

**FIGURE 3.**
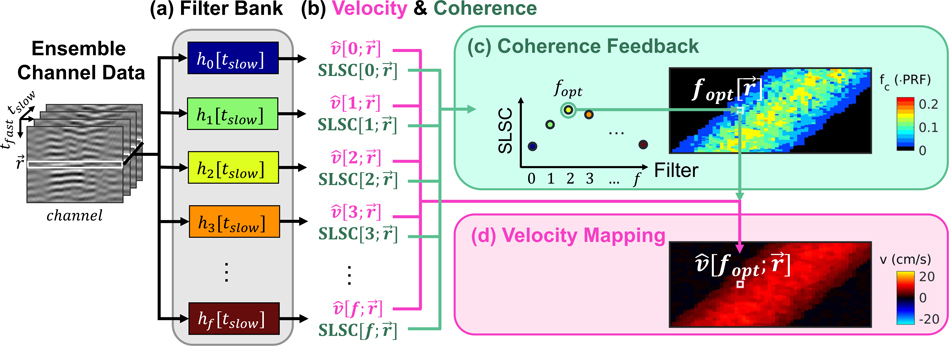
Proposed algorithm for CACF. Processing steps are shown for a single pixel in which (a) ensemble channel data are passed in parallel through a series of clutter filters, *h*_*f*_. (b) spatial coherence and velocity are measured at the output of each filter, (c) the filter *f*_*opt*_ that maximizes spatial coherence is selected, and (d) the velocity measured at the output of *f*_*opt*_ is mapped to the output pixel at $\vec{r}$.

Among many other potential applications of coherence image quality feedback in color flow imaging, of particular interest in this study is its application to adaptive clutter filtering. Fig. 3 outlines an implementation of CACF wherein filters, *h*_*f*_, are dynamically selected in each frame and each imaging pixel within a frame to maximize local spatial coherence and in turn minimize velocity estimation error.

Color flow channel data consisting of an ensemble of slow-time samples are passed through a bank of pre-defined clutter filters, each with a unique frequency response and cutoff frequency. At the output of each filter, spatial coherence and color flow images are formed via Eqs. (8) and (6). Following calculation of spatial coherence and velocity across the outputs of each filter, the spatial coherence at each imaging location $\vec{r}$ is compared across filters *f* to identify the filter that maximizes spatial coherence *f*_*opt*_. The velocity estimate obtained at the output of filter *f*_*opt*_, i.e.$\hat{v}\left[f_{opt},\;\vec{r}\right]$, is subsequently selected and mapped to the appropriate spatial location in the final output image. This operation is repeated across all imaging locations to form a color flow image wherein each pixel represents the output velocity of the clutter filter that maximizes local spatial coherence.

### E. VALIDATION PIPELINE

In this study, CACF was evaluated using simulated channel data generated from the methods described in Section II-A. Adaptive filtering was implemented using a bank containing all 12 PI-IIR filters described in Fig. 2 along with a “no filter” condition, in which velocity and spatial coherence measurements were obtained directly from the raw slow-time signals. At the output of each filter, spatial coherence feedback was measured using SLSC as given by Eq. (6) with corresponding velocity estimates derived via Eq. (8) using a 1λ axial kernel.

#### 1) DEPENDENCE ON SHORT-LAG VALUE AND SIGNAL-TO-NOISE RATIO

The selection of SLSC short-lag value *Q* is associated with a well-known trade-off between resolution and texture, both of which are also affected by the thermal noise level [38]. To study the effect of the short-lag value in the context of color image quality feedback, color flow images formed with CACF were evaluated for a range of SNR_*c,t*_ with *Q* = 1 (lag-one coherence or LOC), 10, and 20, corresponding to roughly 2%, 16%, and 33% of the 60 element transmit aperture.

Resolution effects were characterized by examining the averaged lateral velocity profiles of color flow images for simulated 6 cm/s plug flow over a wide range of SNR_*c,t*_. Under matched noise conditions, spatial coherence texture dependence was evaluated by measuring the standard deviation of velocity estimates and adaptively selected filter cutoffs within a 1.5 × 2 cm region of uniform scatterers translated at 6 cm/s.

#### 2) COMPARISONS TO CONVENTIONAL COLOR FLOW IMAGING

To evaluate the velocity estimation accuracy of CACF, color flow images formed using coherence feedback with *Q* = 10 were compared to reference images formed with conventional color flow processing, i.e. using a single clutter filter with a fixed cutoff frequency. Reference images were formed for conditions with no filtering and filtering at *f*_*c*_ = 0.03 and 0.09 · PRF or (*v*_*c*_ = 2.0 and 5.9 cm/s), serving as representative “low” and “high” cutoff conditions, respectively.

Matched images were generated for plug flow simulations (Fig. 1a) modeling the imaging conditions outlined in Fig. 5, which include 1) 12 cm/s flow with varying incoherent clutter velocity, 2) 12 cm/s flow with varying bulk velocity, and 3) uncluttered flow with varying peak velocity, all of which were simulated with SNR_*c,t*_ = 10 dB. Measurements of velocity estimation bias and standard deviation as quantified by Eqs. (2) were obtained for each condition with average velocity profiles plotted for select examples to highlight differences between coherence-adaptive and conventional filtering. This analysis was repeated for the 50° parabolic flow simulations described in Fig. 1b to evaluate non-uniform flow conditions with both axial and lateral velocity components that more closely model vessels encountered *in vivo*.

## III. RESULTS AND DISCUSSION

### A. SPATIAL COHERENCE OF COLOR FLOW CHANNEL SIGNALS

**FIGURE 4.**
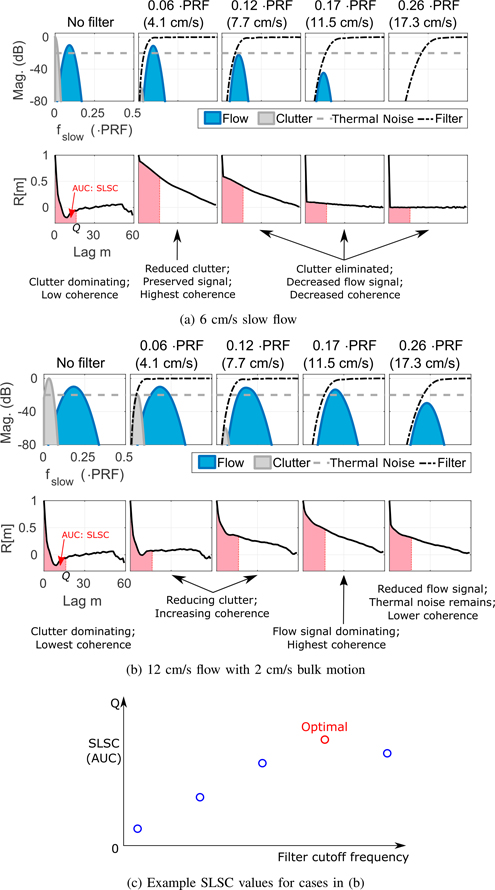
Cartoon examples of slow-time spectra (top) and spatial coherence functions (bottom) for no filter and clutter filters with *f*_*c*_ = 0.06, 0.12, 0.17, and 0.26 ·PRF with corresponding values for *v*_*c*_ in parentheses. Demonstrative examples are shown for (a) 6 cm/s slow flow, wall clutter, and thermal noise and (b) 12 cm/s flow with 2 cm/s bulk motion, wall clutter, and thermal noise. Shaded areas under the curve from lag 0 to *Q* illustrate SLSC values for each case, which are illustrated in (c) for flow conditions in (b). The optimal filter is 0.17·PRF in this case.

To illustrate the relationship between spatial coherence and slow-time spectral content, Fig. 4 shows cartoons of the modeled slow-time spectra containing energy from flow signal, clutter, and thermal noise for select filters along with corresponding plots of the corresponding spatial coherence function. Examples are included for 6 cm/s slow flow (Fig. 4a) and 12 cm/s flow with 2 cm/s bulk motion and off-axis clutter (Fig. 4b). A cartoon scatter plot of SLSC values given the different cutoff frequencies from Fig. 4b is shown in Fig. 4c, where the case with the highest coherence is the recommended cutoff frequency.

**FIGURE 5.**
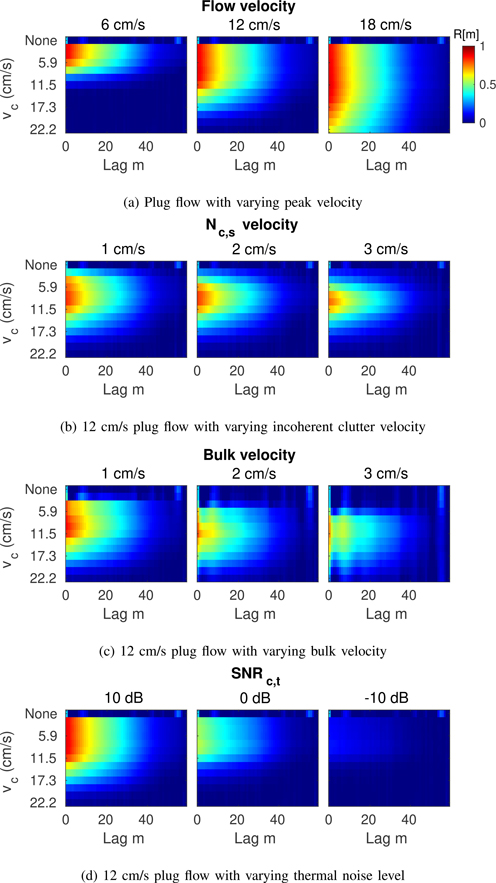
2D colormaps representing the spatial coherence function measured for clutter filters with *f*_*c*_ = 0.03 to 0.34 · PRF or *v*_*c*_ = 2.0 to 22.4 cm/s. Colormaps are shown for varying (a) flow velocity, (b) incoherent clutter velocity, (c) bulk velocity, and (d) thermal noise level. Spatial coherence trends with the relative locations of clutter and blood signal in the slow-time spectrum.

Fig. 5 shows the spatial coherence functions measured at the output of each PI-IIR clutter filter from a 5 × 2 mm region of interest (ROI) located at the vessel center for plug flow under various imaging conditions. Trends in the spatial coherence with filter cutoff are visualized in greater detail in Fig. 5, which shows 2D colormaps of the coherence functions measured across all 12 PI-IIR filters with increasing filter cutoff from top to bottom. The red and blue coloring indicates filters with high and low spatial coherence, respectively. Colormaps are shown across four different imaging conditions: 1) flow with varying peak velocity in the absence of clutter (Fig. 5a), 2) 12 cm/s flow with varying incoherent clutter velocity (Fig. 5b), 3) 12 cm/s flow with varying bulk velocity (Fig. 5c), and 4) 12 cm/s flow with varying thermal noise level (Fig. 5d).

In general, Figs. 4 and 5 show that differences in the relative spatial coherence for different filter cutoffs trend well with the relative locations of weakly coherent clutter and coherent flow signal in the slow-time spectra, suggesting a correlation between measurements of spatial coherence and the relative contribution of clutter and flow signal at the output of each clutter filter. As shown in Figs. 4b, 5b and 5c, where filter passbands are occupied by low frequency clutter from either spatially incoherent noise or weakly coherent off-axis scatter, the spatial coherence is suppressed. Similar trends are observed with thermal noise, which reduces the spatial coherence in filters with high cutoffs that attenuate blood signal, resulting in an effective decrease in SNR_*c,t*_ (Fig. 5a). For sufficiently high levels of thermal noise, coherence is suppressed across all filter outputs as is observed in Fig. 5d as SNR_*c,t*_ is decreased from 10 to −10 dB.

**FIGURE 6.**
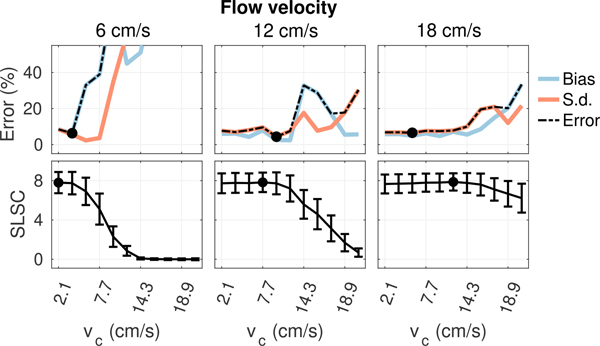
Velocity estimation bias and standard deviation (top) and SLSC (bottom) from matched ROIs in plug flow as a function of filter cutoff with increasing peak flow velocity from left to right. Circles denote the filter cutoffs maximizing coherence and minimizing velocity error. Increasing flow velocity increases coherence and decreases velocity error (circles) at higher cutoffs consistent with the expected shift in the slow-time spectrum of flow signal.

**FIGURE 7.**
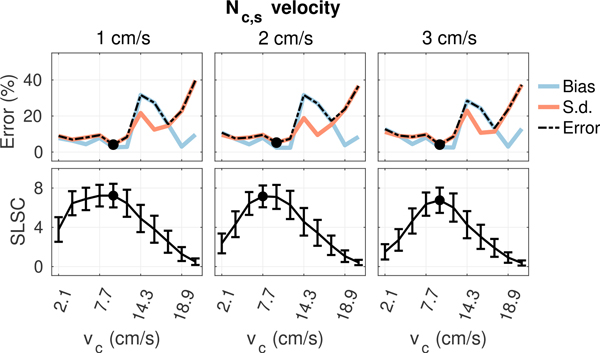
Velocity estimation bias and standard deviation (top) and SLSC (bottom) from matched ROIs in plug flow as a function of filter cutoff for 12 cm/s flow with increasing incoherent clutter velocity from left to right. Circles denote the filter cutoffs maximizing coherence and minimizing velocity error. Increasing clutter velocity decreases the coherence and increases velocity estimation error at the lower cutoffs where energy from incoherent clutter dominates.

**FIGURE 8.**
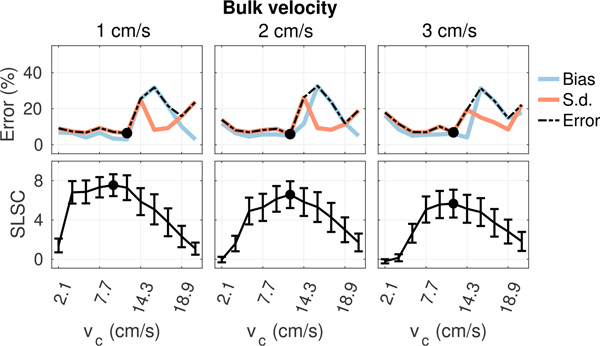
Velocity estimation bias and standard deviation (top) and SLSC (bottom) from matched ROIs in plug flow as a function of filter cutoff for 12 cm/s flow with increasing bulk velocity from left to right. Circles denote the filter cutoffs maximizing coherence and minimizing velocity error. Increasing bulk velocity decreases the coherence and increases velocity estimation error at the lower cutoffs where energy from off-axis clutter dominates.

**FIGURE 9.**
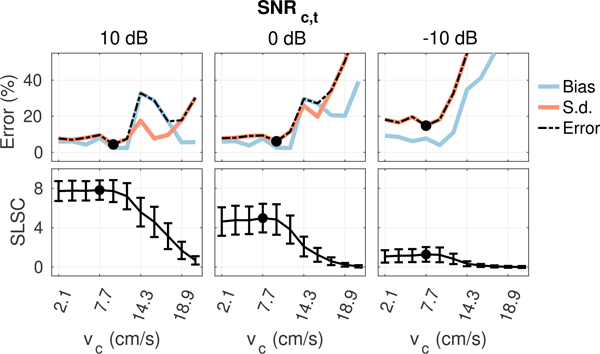
Velocity estimation bias and standard deviation (top) and SLSC (bottom) from matched ROIs in plug flow as a function of filter cutoff for 12 cm/s flow with increasing thermal noise level from left to right. Circles denote the filter cutoffs maximizing coherence and minimizing velocity error. Increasing thermal noise decreases coherence and increases velocity estimation error across all filters.

Figs. 6 to 9 plot measurements of SLSC calculated using *Q* = 10 from the coherence curves in Fig. 5 along with corresponding measurements of velocity bias and standard deviation obtained from the same 5 × 2 mm ROIs used to measure spatial coherence. In general, as the filter cutoff increases, an optimal cutoff is observed where estimation error is at a minimum, followed by a large increase in bias and standard deviation as more aggressive clutter filtering begins to remove flow signal and shape the slow-time spectrum, resulting in an upward shift in the mean slow-time frequency and corresponding overestimation of velocity [39], [40]. At the highest filter cutoffs, velocity estimates become dominated by high variance jitter from thermal noise as the flow signal is completely removed by clutter filtering. Under conditions with acoustic clutter (Figs. 7 and 8), increases in bias are also observed in filters with low cutoffs, consistent with the velocity underestimation that is expected as flow signal is mixed with artifact from slow moving clutter [41]. This effect becomes more obvious as the energy of clutter within the passband of each filter increases with increasing clutter velocity.

As shown in Figs. 6 to 9, trends in bias and standard deviation with filter cutoff are reflected in corresponding measurements of spatial coherence. SLSC values show a clear inverse relationship with velocity estimation error, with coherence increasing as error decreases and the peak coherence occurring close to or directly at the filter cutoff which minimizes error (circles). This relationship is observed consistently in Figs. 6 to 9, supporting the utility of spatial coherence in characterizing color flow image quality.

### B. DEPENDENCE ON SHORT-LAG VALUE AND SIGNAL-TO-NOISE RATIO

**FIGURE 10.**
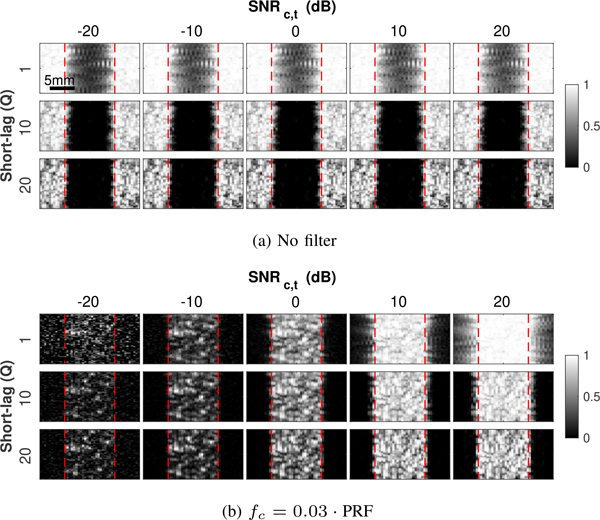
SLSC images created from plug flow channel signals with (a) no filtering and (b) clutter filtering at *f*_*c*_ = 0.03 · PRF for increasing SNR_*c,t*_ from left to right and increasing short-lag value *Q* from top to bottom. Dashed red lines indicate vessel boundaries. Images demonstrate characteristic trade-offs in resolution and texture with changes in SNR_*c,t*_ and *Q*.

Fig. 10 shows a series of spatial coherence images generated using SLSC with *Q* = 1, 10 and 20 for 6 cm/s plug flow for no filtering (Fig. 10a) and clutter filtering with *f*_*c*_ = 0.03 · PRF (Fig. 10b). As with previous studies examining imaging characteristics of SLSC [28], [38], notable differences in the appearance of SLSC images can be observed across short-lag values, with resolution improving and texture noise increasing with higher *Q*. Increases in SNR_*c,t*_, from left to right in Fig. 10, show increasing texture noise for all *Q*. In Fig. 10a, tissue signal dominates in the no filter condition. In Fig. 10b, the clutter filter removes much of the stationary tissue signal, and the effect of increasing thermal noise is more apparent given the lower amplitude of blood scatter.

**FIGURE 11.**
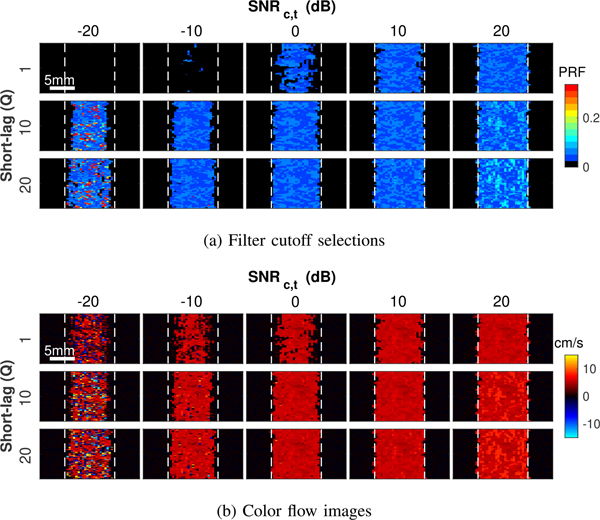
Images of (a) selected filter cutoffs and (b) velocity estimates with CACF for increasing SNR_*c,t*_ from left to right and increasing *Q* from top to bottom. Dashed white lines indicate Vessel boundaries. Similar trade-offs in resolution and texture are observed here as those observed in SLSC imaging.

In CACF, these properties manifest directly in the velocity estimates of output color flow images. Fig. 11 shows maps of the selected filters at each pixel (Fig. 11a) along with the corresponding color flow images formed with coherence-adaptive clutter filtering (Fig. 11b) for different *Q* and SNR_*c,t*_. In general, the same trade-offs in resolution and texture demonstrated in coherence images in Figs. Fig. 10 can be readily observed in the selected filters and velocities in Fig. 11. These observations are detailed in the following sections.

#### 1) IMAGING RESOLUTION

**FIGURE 12.**
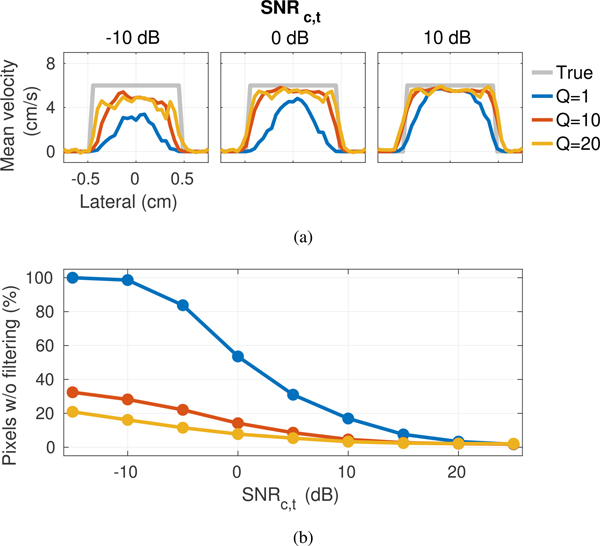
(a) Average velocity profiles with coherence-adaptive clutter filtering for varying *Q* and increasing SNR_*c,t*_ from left to right. Velocity estimates are averaged over a 2 cm axial extent. The ground truth velocity is plotted in gray. (b) Percentage of pixels inside the vessel that select for no filtering as a function of SNR_*c,t*_ and *Q*. The preferential selection of filter conditions containing partially coherent off-axis clutter at low SNR_*c,t*_ and *Q* results in degraded resolution in velocity images generated by coherence-adaptive clutter filtering.

Figs. 12a shows profiles of the average velocity as a function of lateral location generated from color flow images in Fig. 11. For a given SNR_*c,t*_, velocity estimates, particularly at the vessel boundaries, appear to more closely match the ground truth for images formed using higher *Q*. This trend becomes more obvious as SNR_*c,t*_ decreases and differences between the resolution of SLSC for different short-lag values become more significant.

The observed decreases in the resolution of velocity estimates can be directly related to the sensitivity of different coherence metrics to off-axis scatter. As characterized in previous studies [28], [38], off-axis clutter is partially coherent and its coherence as measured by SLSC varies with frequency and distance from the source of interference. For smaller short-lag values, SLSC in locations away from strong scatterers decays at a slower rate compared to SLSC for large short-lag values as demonstrated by the poorer lateral resolution of SLSC images formed with small *Q* in Fig. 10.

In context of CACF, the result of degraded resolution with small *Q* is a preferential selection of the no filter condition at lateral boundaries of the vessel, which contain slow-time signals dominated by partially coherent off-axis clutter of higher amplitude, and thus higher SNR_*c,t*_, relative to underlying backscatter from moving blood. As SNR_*c,t*_ decreases, this preferential selection of filters containing energy from off-axis clutter increases as the relative contribution of incoherent signal from thermal noise becomes greater in filters with blood backscatter than in filters with off-axis clutter, which is of lower intrinsic coherence but higher amplitude than blood.

These trends are well-illustrated by differences in the SLSC images in Figs. 10a and 10b, which show a greater relative increase in the coherence of stationary off-axis clutter that dominates in the no-filter condition (Fig. 10a) over the coherence of moving blood scatter after clutter filtering (Fig. 10b) as *Q* and SNR_*c,t*_ decrease. As illustrated in Fig. 12b, as SNR_*c,t*_ and short-lag value *Q* decrease, coherence-adaptive filtering selects for no clutter filtering for an increasing percentage of pixels inside the vessel. This lack of filtering allows partially coherent signals from off-axis clutter to remain, degrading resolution in velocity images.

#### 2) IMAGING TEXTURE

**FIGURE 13.**
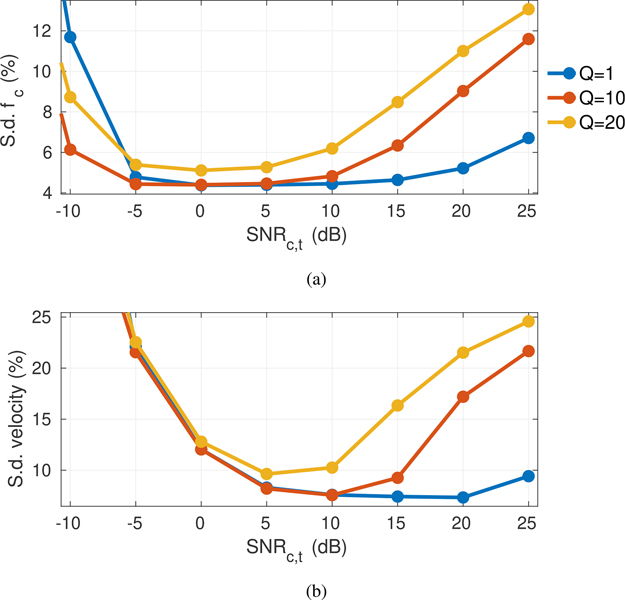
Relative standard deviation measured from (a) selected filter cutoffs and (b) velocities with coherence-adaptive imaging in a 1.5 × 2 cm region of uniform speckle and 6 cm/s velocity for varying *Q* and SNR_*c,t*_. The standard deviation of cutoff frequencies is reported relative to the maximum cutoff available in the filter bank (*f*_*c*_ = 0.34 · PRF). The effects of coherence texture noise and jitter from thermal noise result in increased velocity estimation variance with decreasing SNR_*c,t*_ and increasing *Q*.

Fig. 13 plots the relative standard deviation of filter cutoffs and velocity estimates with CACF for a range of thermal noise levels. At high SNR_*c,t*_, an increase in the standard deviation of selected filter cutoffs is observed with increasing *Q* in Fig. 13a, which is reflected in the resulting velocity estimates in Fig. 13b. Such trends are expected as the peaks and valleys associated with SLSC texture, which become more severe with increasing *Q*, decorrelate between different filter outputs, resulting in an intrinsic variance in the maximum coherence and filter selections at each pixel even under clutter-free conditions with uniform velocity. Consistent with changes in SLSC texture, this variance is also observed to increase with decreasing SNR_*c,t*_. For sufficiently low SNR_*c,t*_, high variance jitter from thermal noise dominates velocity estimates across all clutter filters, resulting in the same increases in standard deviation regardless of *Q*. As shown in Fig. 13b, the combined effects of increased texture at high SNR_*c,t*_ and jitter at low SNR_*c,t*_ result in velocity estimation standard deviation that is minimized at moderate SNR_*c,t*_.

#### 3) DESIGN CONSIDERATIONS

As demonstrated above, the resolution and texture of CACF color flow images can be optimized with careful selection of the short-lag value. While *Q* can be increased to improve resolution, a corresponding increase in standard deviation is expected as texture noise increases. As in SLSC imaging, this dependence on *Q* provides a means to tune CACF based on the requirements of a particular imaging task. In Figs. Fig. 11, a moderate short-lag value (*Q* = 10) is observed to balance between resolution and standard deviation and was therefore chosen for CACF in the subsequent analyses.

Coherence metrics used in the adaptive selection of clutter filters or other flow imaging parameters are not limited to SLSC and can include other measures of spatial coherence such as coherence factor (CF) [26], generalized coherence factor (GCF) [30], phase coherence factor (PCF) [29], and others, which can be similarly adapted to capture changes in the backscatter spatial coherence with changes in color flow sequencing or processing. Such metrics are expected to exhibit similar trade-offs related to the resolution and texture of coherence estimates.

### C. COMPARISONS TO CONVENTIONAL FILTERING IN PLUG FLOW

**FIGURE 14.**
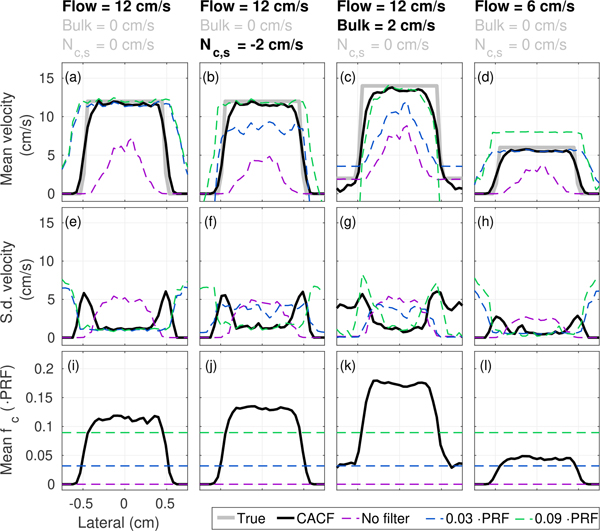
(a)–(d) Average velocity, (e)–(h) standard deviation of velocity, and (i)–(l) average filter cutoff as a function of lateral position with CACF (*Q* = 10), no filtering, and conventional filtering with *f*_*c*_ = 0.03 and 0.09 · PRF for plug flow under various imaging conditions. Averages and standard deviations are calculated over a 2 cm axial extent. The ground truth velocity is plotted in gray. Across all conditions, velocities estimated using CACF closely follow the ground truth with standard deviations on par with conventional filters.

Fig. 14 plots profiles of the average lateral velocity with corresponding plots of standard deviation and average cutoff frequency for simulated plug flow across several example imaging conditions. Under conditions with moving clutter (Figs. 14b and 14c), the presence of clutter energy in the passband of the low cutoff filter (blue) results in underestimation of blood velocity. These velocities can be recovered by suppressing low frequency clutter components with a more aggressive, higher cutoff filter (green). However, this results in a concomitant overestimation of slow flow in Fig. 14d as the filter response begins to remove flow signal and shape the slow-time spectrum. Across all conditions in Fig. 14, velocities without clutter filtering (magenta) show severe underestimation, resulting from the preferential tracking of off-axis clutter over backscatter from moving blood.

Consistent with the analysis in Section II-C, the selection of filters that maximizes SLSC at each pixel with coherence-adaptive clutter filtering is shown to result in velocity estimates that closely follow the ground truth across all conditions examined in Fig. 14. Average velocity profiles (black) demonstrate the simultaneous ability to suppress bias from moving clutter without compromising the accuracy of slow flow measurement. As shown by the average filter cutoffs, such properties are enabled by the appropriate selection of high cutoff filters when low frequencies of the slow-time spectrum are occupied by weakly-coherent clutter to remove slowly moving off-axis scatter or incoherent noise (Figs. 14j and 14k). Meanwhile, under conditions with slow flow, the selection of low cutoff filters helps preserve low velocity signal from coherent blood and/or tissue (Fig. 14l).

Of note is the sensitivity of coherence feedback to clutter that is decoupled with tissue motion in Figs. Fig. 14b where clutter is moving at −2 cm/s and tissue is stationary at 0 cm/s. This represents a failure mode in existing adaptive methods such as adaptive down-mixing where surrogate measures of clutter motion derived from tissue would more closely reflect the velocity of higher amplitude stationary tissue over that of moving clutter, resulting in minimal adaptive feedback and little change to the local filter characteristics.

Interestingly, standard deviations in Fig. 14 demonstrate that the dynamic selection of clutter filters, which varies among different vessels and individual pixels, is not associated with any significant increases in velocity estimation variance. Figs. 14e to 14h reveal standard deviations in CACF on par with those obtained in conventional filtering, tracking closely with the best-case fixed cutoff filter in each condition. Increases in standard deviation are, however, observed at the lateral boundaries of the vessel. As shown in color flow images in Fig. 11, this is likely a resolution effect related to variance in the selection between filters that track either moving blood or stationary tissue as signals from the two types of scatterers begin to mix. The simultaneous measurement of both blood and tissue motion is a unique ability enabled by CACF. As shown in Figs. 14a to 14d, such a property results in velocity estimates inside, outside, and at the boundary of the simulated vessel, which more closely reflect the ground truth velocity profile and better preserve vessel boundaries that are otherwise blurred by conventional clutter filters – often referred to as “blooming” [42].

### D. COMPARISONS TO CONVENTIONAL FILTERING IN PARABOLIC FLOW

**FIGURE 15.**
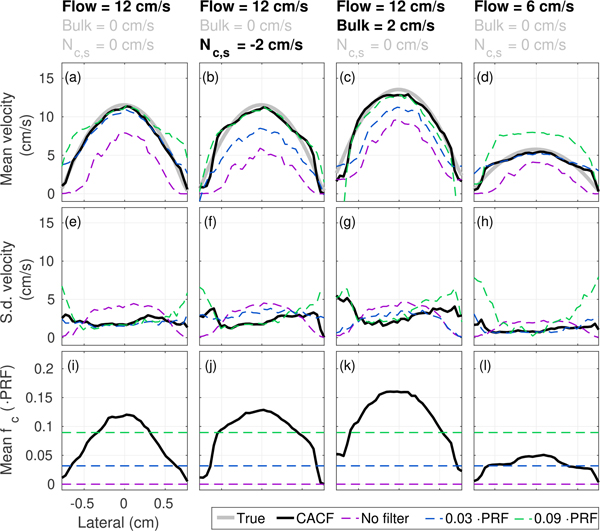
(a)–(d) Average velocity, (e)–(h) standard devation of velocity, and (i)–(l) average filter cutoff as a function of lateral position with CACF (*Q* = 10), no filtering, and conventional filtering with *f*_*c*_ = 0.03 and 0.09 · PRF for 50° parabolic flow under various imaging conditions. Averages and standard deviations are calculated over a 2 cm axial extent. The ground truth velocity is plotted in gray. Variations in flow and clutter velocity across different vessels and regions of the same vessel are reflected in the selected filter cutoffs and output velocities with CACF.

These same velocity and clutter conditions were examined for non-uniform, 50° parabolic flow in Figs. Fig. 15. As in the plug flow examples, average velocity profiles in Figs. Fig. 15 demonstrate the same ability for CACF to maintain accurate velocity estimation under various cluttered conditions with parabolic flow. This is reflected in the selected filter cutoffs, which trend with local clutter and flow velocities to adaptively suppress moving clutter (Figs. 15b and 15c) without significant attenuation of slow flow signal (Fig. 15d). Likewise, improvements in accuracy are coupled with low standard deviations that closely match those of the best-case fixed cutoff filter.

It should be noted that across the examples in Fig. 15, SLSC feedback selects for a wide range of filter cutoffs, going from no filtering to a maximum average cutoff frequency approaching *f*_*c*_ = 0.17 · PRF. Such a result suggests that conventional filters with fixed frequency responses are largely insufficient to fully optimize the spatial coherence and therefore the velocity estimation accuracy across different vessels and even different regions within the same vessel.

**FIGURE 16.**
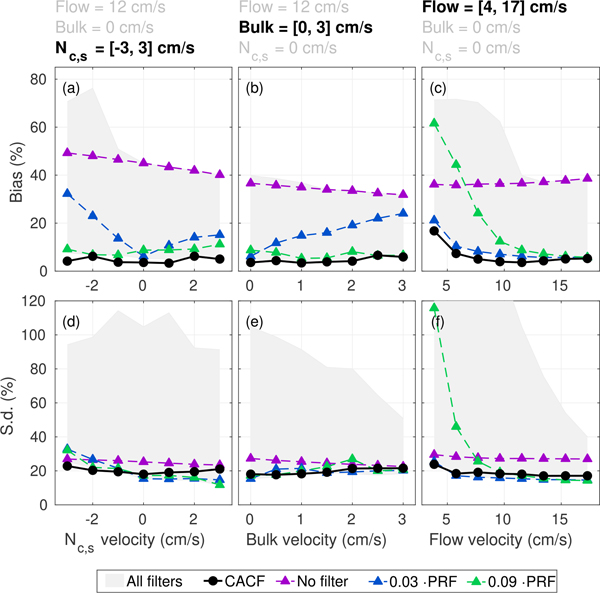
Summary statistics capturing the (a)–(c) bias and (d)–(f) standard deviation of velocity across the 50° parabolic vessel for different incoherent clutter, bulk, and peak flow velocities with CACF (*Q* = 10), no filtering, and conventional filtering with *f*_*c*_ = 0.03 and 0.09 · PRF. The range in bias and standard deviation measured across all filters in the filter bank is shown in shaded gray. CACF minimizes bias across a range of different flow and clutter conditions without comprise to the variance of output velocity estimates.

Measurements of velocity estimation bias and standard deviation from simulations of 50° parabolic flow are summarized in Fig. 16, which describes the averaged statistics across the entire vessel calculated via Eqs. (4) and (5) over a range of incoherent clutter, bulk, and peak flow velocities. Figs. 16a to 16c show measurements of bias that are consistently lower in CACF compared to conventional filtering. These results are consistent with observations made from the example velocity profiles in Figs. 14 and 15 and similarly demonstrate the underestimation of flow velocity that is expected in low cutoff filters in the presence of slow-moving clutter and the overestimation of flow velocity that is expected in high cutoff filters under conditions with low velocity flow. For the low cutoff filter in Figs. 16a and 16b (blue), underestimation due to moving clutter increases as clutter velocity and the contribution of clutter energy to the slow-time spectrum increases. For the high cutoff filter in Fig. 16c (green), velocity overestimation increases as flow velocity decreases and greater amounts of flow signal are attenuated. In contrast, CACF (black), which dynamically adjusts filter cutoff based on clutter and flow slow-time spectral content, is able to maintain accurate velocity estimation with minimal bias across all conditions. In the presence of clutter from spatially incoherent noise or bulk tissue motion (Figs. 16a and 16b), CACF is able to effectively suppress bias introduced by low frequency clutter. While under clutter-free imaging conditions, the method helps to increase dynamic range and to minimize error in the measurement of slow flow (Fig. 16c).

Corresponding measurements of standard deviation are shown in Figs. Figs. 16d to 16f and demonstrate that trends related to estimation variance in Figs. Figs. 14 and 15 generalize to a range of imaging conditions. Across the clutter and flow velocities examined in Fig. 16, standard deviations with CACF fall well within the range (gray) and close to the lower bound of standard deviations achieved with conventional filtering. Together, these results demonstrate promise for coherence feedback to provide effective pixel-wise selection of clutter filters without significantly increasing variance.

### E. LIMITATIONS AND FUTURE WORK

While simulations in this study attempt to model a wide range of flow velocities and clutter realizations, it is impossible to fully capture the diversity of imaging conditions that are present *in vivo*. Additional studies are needed to examine the performance of this technique on real data obtained from phantoms and *in vivo* vessels in order to more formally evaluate its clinical feasibility.

The model used to simulate off-axis clutter using bulk motion of the block of scatterers was restricted to movements in the direction of the blood flow. Bulk motion opposing the direction of flow would impose significant challenges to CACF. These conditions should be addressed in a future study.

Models for flow and clutter in this study exclusively include those in which flow and clutter content can be readily separated using conventional high-pass filters. Under realistic imaging conditions, the spectral energy of clutter is not necessarily restricted to lower frequencies, and under conditions where flow signal and clutter have significant spectral overlap or when clutter is of higher velocity than blood, this implementation of CACF will likely break down.

The selected filter cutoffs seem to correlate much more with blood velocities than clutter velocities. Spectrally broad flow within a sample volume, such as aliased or turbulent flow, would likely lead to similar coherence values among filters, leading to increased variance in velocities, which itself results from increased variance in filter selections. For example, in flow with high slow-time blood signal spectral bandwidth, the SLSC will be largely constant for a wide range of filter cutoffs yielding significantly different velocity estimates. The results presented here shown do not address this concern, as they include either plug flow or parabolic flow in a vessel that is large compared to the sample volume, with relatively low blood velocities. Increasing the range of velocities to above the Nyquist limit and considering these additional conditions should be addressed in future work.

A potential solution to such failure modes may be to incorporate other filter types, such as band-pass or matched filters, beyond the 12 high-pass filters used in this study. In the same way that spatial coherence feedback can be applied to adaptive select FIR or IIR clutter filters, a similar pipeline could adaptively identify eigen-components in eigen-based filtering or polynomials in regression filtering. The extension of coherence feedback to these higher-order techniques has potential to fully eliminate any dependence on clutter frequency and magnitude and provide a means to separate slow-time signals based solely on their spatial coherence.

Along these lines, future studies should explore the dependence of CACF on the composition of filters in the filter bank, including the number and diversity of available filters. Such analyses may inform the design of more efficient implementations of CACF, which are needed to realize this technique in practice.

In addition to reducing the number and complexity of the filter bank, techniques to reduce the total number of coherence calculations can be leveraged to further improve computational efficiency. One approach would be to sparse the number of SLSC calculations along the channel, slow-time, or spatial dimensions. Such a strategy shows promise particularly in applications of spatial coherence for image quality feedback where resolution requirements are generally less stringent than in coherence imaging. The linearity of FIR and IIR filtering operations furthermore shows promise for more streamlined formulations to improve the tractability of performing multiple filtering operations and coherence calculations in parallel.

In this work, coherence-adaptive clutter filtering was only compared to a standard single clutter filter with certain cut-off frequencies. Future work should compare this novel method to other adaptive methods of clutter filtering, namely the previously mentioned eigen-based and tissue motion measuring filters. It is an open question as to how these existing methods perform relative to the results shown here.

Finally, it should be noted that the proposed method represents a general tool to optimize clutter filtering in any type of motion estimation task, not limited to color flow imaging. As such, CACF is readily adaptable to methods such as vector flow imaging and spectral Doppler, which are similarly affected by clutter filter selection.

## IV. CONCLUSION

In this study, we demonstrated the application of spatial coherence image quality feedback for the adaptive selection of clutter filters in color flow imaging. Building off intuition gained from studies exploring spatial coherence in conventional B-mode imaging, we explored the relationship between color flow image quality and measurements of SLSC from clutter filtered channel data. This relationship was leveraged to devise a novel method for coherence-adaptive clutter filtering, or CACF, designed to select clutter filters on a pixel-wise basis to maximize local spatial coherence and velocity estimation accuracy. CACF was evaluated across a wide range of simulations modeling different vessels, clutter realizations, and flow rates, all of which demonstrated the ability for CACF to reduce velocity estimation bias from clutter relative without significantly increasing velocity estimation variance or compromising the accuracy of measurements in slow flow. Together, findings in this study show promise for coherence feedback to reduce the operator dependence of color flow imaging and enable the automated selection of clutter filters without the *a priori* assumptions of clutter magnitude and frequency required by existing methods for adaptive filtering.
